# The Efficacy of Strength Exercises for Reducing the Symptoms of Menopause: A Systematic Review

**DOI:** 10.3390/jcm12020548

**Published:** 2023-01-09

**Authors:** Ana María Capel-Alcaraz, Héctor García-López, Adelaida María Castro-Sánchez, Manuel Fernández-Sánchez, Inmaculada Carmen Lara-Palomo

**Affiliations:** Department of Nursing, Physical Therapy and Medicine, University of Almeria, Road Sacramento s/n, 04120 Almeria, Spain

**Keywords:** menopausal, climacteric syndrome, resistance training, therapeutic exercise, physiotherapy

## Abstract

Background: The aim of this systematic review was to determine whether strength exercises improve the symptoms of menopause and to provide an update on the most recent scientific evidence on the type and regimen of exercise that help reduce the symptoms. Methods: An electronic search of scientific databases was performed from 2015 to 2022. Randomized clinical trials that analyzed the effects of strength exercises versus other types of interventions, considering all the outcome measures of interest, were included in this review. Results: We found 5964 potential articles. After applying the selection criteria, we selected 12 of the articles. The studies compared strength exercises versus other therapies or compared strength exercises versus no intervention in one of the groups. The results showed improvements in the strength of the legs and pelvic floor, physical activity, bone density, metabolic and hormonal changes, heart rate and blood pressure and a change in hot flashes. Conclusions: There is evidence that strength exercises can be beneficial for improving strength, physical activity, bone density and hormonal and metabolic levels. In terms of the appropriate type of strength training, the evidence is still unclear given that the same benefits are achieved by various types of exercises.

## 1. Introduction

Menopause is defined as the condition of the permanent cessation of menstruation, determined retrospectively after 12 consecutive months of amenorrhea, with no pathological causes [1]. Menopause typically occurs between the ages of 45 and 55 years and is due to the cessation of estrogen production due to stimulation by the follicle-stimulating and luteinizing hormones as a result of ovarian follicular atresia [2,3,4].

The set of menopausal symptoms is known as climacteric syndrome, and these symptoms are highly varied [5]. The most common of these symptoms include hot flashes, fatigue, increased cardiovascular risk, urogenital problems, sexual dysfunction, sleep disorders and mood disorders [6]. According to certain studies, the most common symptom is hot flashes, which affect 75% of menopausal women and can persist from 4 to 5 years (in some cases lasting more than 10 years or for life) [6,7,8]. In addition to these problems, the reduction in estrogen can cause bone mass loss, resulting in osteoporosis, whose prevalence increases with the woman’s age [9].

There are various drug treatments for fighting the symptoms of menopause [10,11]. Hormone therapy with estrogens or combined with progestogens is one of the most widely used treatments for hot flashes; however, it has been observed to cause adverse effects such as breast cancer and cardiovascular problems [12,13,14].

Physical exercise can be an alternative to drug treatment [15]. Studies have shown that exercise increases muscle strength and bone mineral density, improves motor control, equilibrium and muscle coordination and consequently reduces the risk of falls and improves the quality of life [14,16]. Progressive strength exercises have been shown to improve physical ability and increase lean mass in older adults [17], which could be extrapolated to the menopausal stage.

Given that active therapeutic exercise has been shown to be beneficial for various conditions, the aim of this systematic review was to determine the effects produced by strength exercises on the symptoms of climacteric syndrome in menopausal women and to provide an update on the most recent scientific evidence on the exercise type and regimen that have the greatest effect on these symptoms.

## 2. Materials and Methods

### 2.1. Protocol and Registry

This systematic review has been performed following the recommendations of the Preferred Reporting Items for Systematic Reviews and Meta-Analysis (PRISMA) [18]. A systematic review on the effects of strength exercises for reducing the symptoms of menopause was carried out using the Assessing the Methodological Quality of Systematic Reviews (AMSTAR) 2 tool [19]. Systematic review registration: www.crd.york.ac.uk/PROSPERO. PROSPERO registration number: CRD42022378721.

### 2.2. Study Design

We conducted a systematic review of the scientific literature by searching databases for published studies on the efficacy of strength exercises for reducing the symptoms of menopause. This was followed by a critical analysis of the scientific literature retrieved from the literature search.

### 2.3. Source Data and Search Strategy

We conducted a literature search from April 2020 to December 2022 in the following databases: PUBMED, PEDro, Web of Science and Cochrane.

The clinical issues for the critical analysis are based on the PICO format [20] (Patient, adults with menopausal symptoms; Intervention, strength exercises; Comparison, no intervention or other therapies; Outcome, valid and reliable measures that evaluate the menopausal symptoms, which were not specified because all outcome measures were considered of interest).

For the search, we combined the Boolean operator AND with the following MeSH terms and keywords: “postmenopausal”, “strength exercise”, “postmenopause” [MeSH], “muscle strength” [MeSH], “menopause” [MeSH] and “exercise training” [MeSH]. We limited the search in each database to articles in which the terms appeared only in the title or abstract of the article. We eliminated from the selection duplicate articles identified in the multiple database searches and established the publication date limits (from 2015 to 2022).

### 2.4. Study Screening: Inclusion and Exclusion Criteria

Studies included in the review had to meet the following inclusion criteria: (1) randomized clinical trial (RCT) published in English since 2015; (2) in which the effects of strength exercises were analyzed; (3) compared to other types of interventions or no intervention; (4) on relieving menopausal symptoms; (5) in women in the menopausal stage.

The exclusion criteria were: (1) protocols for conducting RCTs, systematic reviews or quasi-experimental studies or those in regard to a case; (2) studies in which the sample included patients with a pathological cause for the menopause; (3) studies in which the strength exercises were not the main treatment or were combined with several therapies, which precluded assessing the effectiveness of the treatment by itself.

We restricted the studies’ eligibility by language but not by publication status. We allowed for studies with co-interventions if these were comparable between the intervention groups. All results had to have been obtained using valid and reliable instruments.

### 2.5. Data Extraction

The authors (A.M.C.-A. and A.M.C.-S.) individually reviewed the titles and abstracts of the articles encountered to identify potential valid studies and to review the full text. The full text of the selected studies was assessed independently to determine whether it met the selection criteria and could be added to the review. Disagreements were resolved by consensus or by consulting a third author (I.C.L.-P.). We also reviewed the references that presented other reviews and articles, in case any of them could be included in the study and had gone unnoticed in the online search. We completed the search using the snowball method.

The studies’ main characteristics (e.g., sample size, mean age, intervention program, variables and relevant results) were extracted into an Excel spreadsheet. Then, a general table was made on the characteristics of each study and the synthesis of the evidence. One author (A.M.C.-A.) carried out an analysis of the type and volume of exercise, the materials used and its supervision and made a table of the characteristics of the strength exercise interventions.

### 2.6. Risk-of-Bias Tool

Studies that met the inclusion criteria were assessed by two authors (H.G.-L. and M.F.-S.) according to the Cochrane risk-of-bias tool [21]. The clinical trials were rated as being of low risk, high risk or uncertain risk with regard to seven items: random sequence generation, allocation concealment, blinding of participants and personnel, blinding of the outcome assessment, incomplete outcome data, selective reporting and other biases.

## 3. Results

### 3.1. Search Results

**Figure 1:**
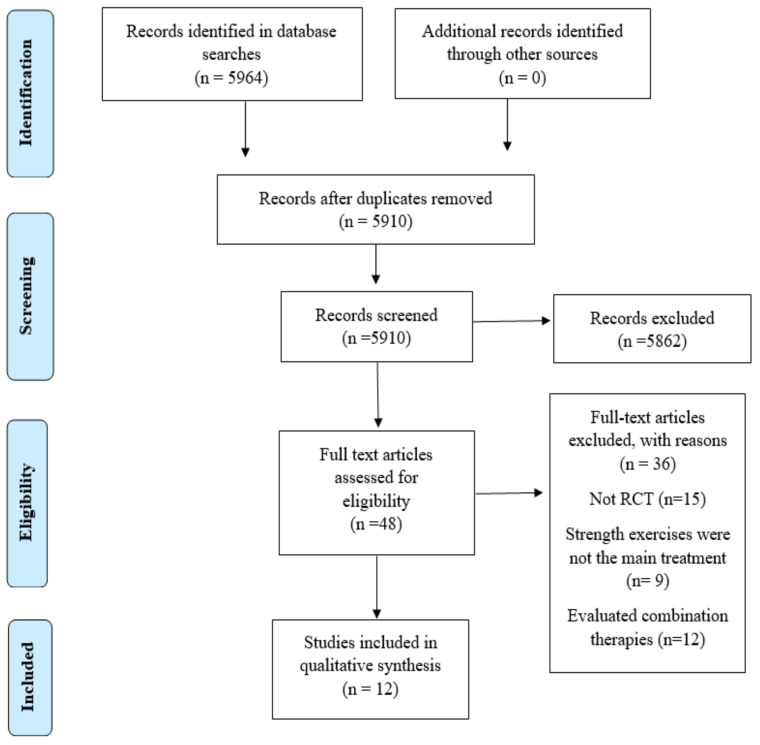
Eligibility and data synthesis: PRISMA flow diagram.

The initial search identified 5964 potential articles, 5862 of which were excluded based on their title and abstract; 54 were duplicates, leaving 48 articles for the full text review. After applying the inclusion and exclusion criteria, we rejected 36 articles, leaving 12 for the qualitative synthesis. Figure 1 shows the PRISMA flow diagram for the study selection process.

### 3.2. Characteristics of the Included Studies

We included 12 RCTs covering 817 participants [7,15,22,23,24,25,26,27,28,29,30,31]. Two of the RCTs were published in 2015 [15,24], two in 2016 [25,26], one in 2017 [27], two in 2018 [28,29], three in 2019 [7,30,31] and two in 2020 [22,23]. The study with the smallest sample size had 20 patients [23], and the study with the largest sample had 194 patients [15].

Four of the studies were conducted in Brazil (with a total of 177 patients) [24,25,26,30], three were conducted in Australia (with a total of 246 patients) [28,29,31], one was conducted in Norway (with 194 patients) [15], one was conducted in Finland (with 80 patients [27], one was conducted in Sweden (with 58 patients) [7], one was conducted in the United Kingdom (with 42 patients) [22] and one was conducted in South Korea (with 20 patients) [23].

Seven of the selected articles did not specify the study chronology [15,22,24,25,27,30,31], while five did [7,23,26,28,29]. Three of these five studies also reported the chronology of the patient recruitment process and the chronology of the follow-up period [23,28,29]. The studies’ recruitment period took place between July 2012 and November 2013 [26], between May 2014 and November 2015 [29], between March 2013 and September 2014 [28], between November 2013 and October 2015 [7] and between February 2018 and March 2018 [23]. The follow-up period for three of the studies that mentioned the period was August 2016 [29], December 2016 [28] and July 2018 [23]. All studies were published in English [7,15,22,23,24,25,26,27,28,29,30,31].

All studies were performed with menopausal women, without specifying the participants’ race, ethnic origin or educational level, and all studies reported the participants’ mean age (mean age for all study participants, 62 years). The study with the youngest sample was that of Berin et al. [7], with a mean age of 55.3 years, and the study with the oldest sample was that of Son et al. [23], with a mean age of 67.7 years.

All studies described the recruitment method, except that of Nunes et al. [30], and the methods were highly heterogeneous. The participants were included from the department of orthopedic surgery and the emergency department of a university hospital [15], through a previous study [24,31], from a neighborhood association [24], from a university extension project “Attention to women’s health” [26], through advertisements from referrals by Victorian gyms, through physicians and allied healthcare practitioners, from national clinics, from pharmacies [7,22,27,28,29], from sports clubs and women associations [28] and through clinical referrals in health centers [23].

All studies employed strength exercises versus other types of interventions such as home exercises [24,29,31]. One study involved a warmup [24], a hike with active exercises and resistance exercises of the abdomen and extremities, along with a final relaxation stage. Two studies consisted of walking, low-resistance exercises and stretching [29,31]. One study consisted of mobility exercises, isometric stretching and strength exercises [26], and another study consisted of simulated exercise and stretching [28]. Two studies compared three groups: one group with high-volume strength exercise, one with low-volume exercise and one with stretching [25,30]. One of the studies compared the same leaping exercise, but each group used a different leg [22]. There were also four studies that had a non-intervention group [7,15,23,27].

The sessions were highly heterogeneous: three weekly sessions in four studies [7,15,23,27], two weekly sessions in four studies [24,26,29,31], one daily session in one study [22], two sessions a week for the control group and three sessions a week for the experimental groups in two studies [25,30] and no specified treatment session regimen in one study [28].

Ten studies conducted a follow-up at two separate times (start and end) [7,23,24,25,26,27,29,30,31]. Only two studies conducted the follow-up at three separate times [15,29]: at the start and at 6 and 12 months of the intervention in the study by Hakestad et al. [15] and at the start and at 3 and 12 months of the intervention in the study by Ganderton et al. [28]

There was no attrition in nine of the studies [7,15,22,23,26,28,29,30,31]. The study by Bittar et al. [24] had 14 dropouts in the experimental group and 12 in the control group, the study by Prado-Nunes et al. [25] had 6 dropouts, and the study by Multanen et al. [27] had 4 dropouts.

Table 1 explains each study by presenting a synthesis of evidence for strength exercises on symptoms of postmenopausal women.

### 3.3. Characteristics of the Strength Exercises in the Included Studies

In seven of the included studies, the strength exercise intervention was performed with multi-joint exercises, both in the arms and legs. Five studies employed fitness machines [7,25,29,30,31], one study employed a weighted vest [15] and two studies employed elastic bands [23,24]. A single study conducted strength training of the pelvic floor muscles through virtual reality performing pelvic movements, maintaining trunk control and stabilization and abdominal activation [26]. Two studies employed the leap as the strength exercise [22,27], and one study focused on the strength training of the legs (gluteus medius, quadriceps and calves) [28]. All interventions had an initial warmup stage to prevent injuries. All studies were supervised except for the study by Hakestad et al. [15].

The session times varied significantly. The studies by Prado-Nunes et al. [25] and Ganderton et al. [28] did not specify the duration, but the remaining studies did. The duration varied from 90 min of training in the intervention group in the study by Nunes et al. [30] to 8–9 min of training in the study by Hartley et al. [22]. 

Table 2 shows the characteristics of the strength exercise interventions included in this review. The table shows the type of exercise, the material employed, the session time, the number of series, the number of repetitions, the time of repetitions and whether the exercise was supervised.

### 3.4. Methodological Quality of the Included Studies

The results of the Cochrane risk-of-bias tool [21] did reveal that seven articles were of low risk [7,15,22,23,26,27,28], two were of high risk [25,30] and three were of unclear risk [24,29,31]. Nine articles were of low risk [7,15,23,24,26,27,28,29,31] and three were of high risk of bias in “random sequence generation” [22,25,30]. Seven articles were of low risk [7,15,22,23,26,29,31] and five were of high risk of bias in “allocation concealment” [24,25,27,28,30]. Six articles were of low risk [15,22,23,26,27,28] and the other six studies were of high risk of bias in “blinding of participants and personnel” [7,24,25,29,30,31]. Four articles were of low risk [7,15,22,28] and eight studies of were high risk of bias in “blinding of outcome assessment” [24,25,26,27,29,30,31]. All studies were of low risk of bias in “incomplete outcome data”, except for one of them [31], which was of high risk of bias. Finally, all articles were of low risk of bias in “selective reporting” and “other bias”. The results of applying the Cochrane risk-of-bias tool can be observed in Table 3.

### 3.5. Results of the Included Studies

#### 3.5.1. Strength

Leg extension strength was measured in three clinical trials [15,25,29], all employing a dynamometer for the measurement, but the results were statistically significant (*p* < 0.001) in favor of the experimental group in only one study [29]. Similarly, one study [27] obtained significant results (*p* < 0.01) in bone strength and knee cartilage using a scanner. In addition, only one study [29] found significant improvements in favor of the experimental group (*p* < 0.001) in muscle performance, as measured with the maximum leap in a force plate.

Only one study [26] measured the strength of the pelvic floor muscle using palpation and a dynamometer, and there were only significant differences in relation to muscular endurance in favor of the experimental group (*p* = 0.003).

#### 3.5.2. Physical Activity

The physical activity level or tolerance was measured using various scales such as the Physical Activity Scale for the Elderly (PASS) [15], the Bone-Specific Physical Activity Questionnaire (BPAQ) [29], the International Physical Activity Questionnaire (IPAQ) [7] and body movement monitoring instruments [27]. Only three [7,27,29] of the four studies [7,15,27,29] that evaluated this variable found statistically significant differences.

#### 3.5.3. Bone Density

Four studies measured the participants’ femoral bone density using X-ray absorptiometry [15,22,24,29], but only in two studies were there statistically significant differences in favor of the experimental group [22,29]. One of the studies [31] also measured the magnitude of the kyphosis dorsalis using X-ray absorptiometry and an inclinometer, and there was a significant result (*p* = 0.031) in favor of the experimental group.

#### 3.5.4. Hormonal and Metabolic Changes

Three studies took blood samples to compare hormonal changes or changes in metabolic and inflammatory factors, as well as growth hormone, insulin-like growth factor 1, dehydroepiandrosterone sulfate, testosterone and cortisol levels [23,25,30]. There were only significant results in two studies [23,25]. On the other hand, heart rate [7] and blood pressure [23] were only measured by one study, finding statistically significant results in the improvement of heart failure [7] and hot flashes [23].

#### 3.5.5. Other Variables Analyzed

No statistically significant differences were found in the results of the studies that evaluated quality of life [15,27,28], anthropometry [15,23,25], food intake [29,30], dynamic balance [15], dominant heel [29], pain in the gluteal tendon [28] and the presence of osteoarthritis [22].

## 4. Discussion

### 4.1. Summary of Evidence

We set out to conduct a unique, up-to-date review on the impact of strength exercises on menopausal women. We found 12 published RCTs that evaluated the efficacy of strength exercises for these patients, with a total of 817 patients.

In general, we found that interventions with strength exercises as the sole intervention generate, with moderate-quality proof, significant improvements in strength, physical activity, bone density and hormonal and metabolic changes in menopausal women compared to an inactive control group, placebo or other interventions.

For example, when comparing these types of exercises with unsupervised home exercises [24,29,31], the results were in favor of the strength exercise group for improving aspects such as increased lean mass, increased femoral bone density and reduced kyphosis. As shown by Watson et al. [31], there was no change in the vertebral fracture classification, i.e., it remained stable in the strength exercises group but not in the home exercise group, in which a participant experienced a vertebral wedge fracture.

Simulated exercise does not appear to have benefits for menopausal symptoms, such as hip dysfunction and gluteal tendon pain. In contrast, the isometric exercises of the gluteus mediums, quadriceps and calves seem to produce benefits for these symptoms [28].

When comparing the low-volume strength exercises with the high-volume exercises, the results favor the high-volume exercises, both for reducing cholesterol and for muscle performance and lean mass [25,30]. Moreover, the comparation between stretching exercises and strength exercises (both low- and high-volume) shows that stretching exercises have no benefits for muscle performance and for the hormonal responses of menopause [26].

Leap exercises seem to improve the bone density in the femoral neck, but there was no effect on the knee cartilage composition [22,27]. When comparing a group that performed some type of strength exercise against one that did not undergo any intervention [7,15,23,27], there were no improvements for the non-intervention group. Berin et al. [7] showed that strength exercises decrease the heart rate and hot flashes. Son et al. [23] reported an increase in estradiol, growth hormone, insulin-like growth factor 1 and dehydroepiandrosterone sulfate levels and a reduction in systolic blood pressure, total body mass, body mass index and body fat percentage. However, the change in blood pressure was not maintained over time. At the start, the results of the study by Hakestad et al. [15] favored the strength exercises intervention group due to the improvement in aspects such as absolute mass, percentage body fat, dynamic balance, walking ability, physical activity level and quality of life. However, at 1 year of follow-up, the results between the groups were similar, which could be because, if the exercises are not practiced assiduously, the benefits are not maintained.

Several factors should be considered when interpreting our findings for clinical recommendation and implementation. Each study reported a clinically relevant improvement in favor of the experimental group with strength exercises. The efficacy of strength exercises is unclear because the qualitative analysis was hampered by the small number of studies that included the same outcome measures and a follow-up beyond the period immediately following the intervention.

### 4.2. Agreements or Disagreements with Other Studies or Reviews

Strength exercises and their effect on menopausal women have been briefly described in the literature, but this is the only review that considered all the general benefits that these exercises provide women with climacteric syndrome. This is because the main difference between the present study and previous analyses might be the more careful screening of various outcome variables.

Other reviews that have attempted to demonstrate the efficacy of strength exercises have also faced the challenge of only finding articles of low methodological quality, and as a result, a small number of articles are included in the meta-analysis. In 2019, the review by Daly et al. [9] concluded that strength exercises are effective for improving aspects such as fracture risk, but the benefits depend on the exercise type and regimen. Despite this, our results are consistent with those of Fernandes et al. [32], which show that intense exercises (70% to 90% of one max repetition) performed at a frequency of two to four times a week appear to be effective in the improvement of muscle strength, bone density and physical function. Our results indicate that exercises performed two to three times per week for more than 4 months are statistically significative in terms of improving strength [24], bone mineral density [29] and hormonal and metabolic changes [24]. Likewise, Daly et al. [9] confirmed that progressive resistance exercises performed more than twice per week are beneficial for menopausal symptoms, but the exercises were highly heterogeneous, and it is unclear which regimen provides the greatest benefits.

A recently published article [33] also obtained outcomes that tend towards the statistical significance of strength exercises for the improvement of bone mineral density. Our article correlates with this article, as we observed low–moderate evidence of an impact of dynamic resistance exercise on bone mineral density changes in postmenopausal women. However, in summarizing the few other meta-analyses [34,35,36,37] that focus on the effect of resistance exercises on the bone mineral density of the proximal femur, the effect sizes vary considerably.

Likewise, Atapattu et al. [38] demonstrated the beneficial effects that exercise provide in menopausal women regarding increased vagal tone, the influence of stress hormones and parasympathetic activation and the activity of the thermoregulatory center. However, other studies did not find sufficient evidence to determine the effects of exercise on hot flashes and night sweats [39,40,41,42,43]. These results were similar to our findings, since only the study by Berin et al. [7] evaluated this variable.

### 4.3. Limitations and Strengths of the Study 

This review is limited by the small number of studies found with a high methodological quality, the presence of the same risk of bias in all studies, the lack of the blinding of the therapists and patients, the heterogeneity in the sample sizes, the type of therapeutic interventions, the variables analyzed, the secondary results and follow-up and, lastly, by the low number of studies performed to date. The effects of these types of exercises should be interpreted with caution because the protocols were highly heterogeneous, both in the type of exercise performed (e.g., repetitions, series, duration) and in the accessory material employed.

Future studies should have larger samples and greater homogeneity in terms of the type of exercise employed to thereby come to firmer conclusions regarding the benefits these exercises provide.

Lastly, given that this review considered all outcome measures to determine the beneficial effects of strength exercises, we consider that our study provides new and current general knowledge on the beneficial effects of strength training on the symptoms of menopausal women.

## 5. Conclusions

Considering the studies encountered, strength exercises can be beneficial for improving menopausal symptoms that affect muscle performance in general, physical activity, bone density and hormonal and metabolic responses such as heart rate, blood pressure and hot flashes. In terms of the appropriate type of strength training, the evidence is still unclear given that the same benefits are achieved by various types of exercises and with various accessory methods.

## Figures and Tables

**Table 1:** Synthesis of Evidence for Strength Exercises on Symptoms of Postmenopausal Women.

Study	Country	Funding	Sample Size	Mean Age	Attrition	Diagnosis	Duration	Intervention	Outcome Measures	Results
Berin, E. et al., 2019 [7]	Sweden	Yes	N = 58 EG = 29 CG = 29	55.3	NR	Postmenopausal women ≥12 months amenorrhea	15 weeks	EG = R (three times/week, with 225 min as the maximum exercise per week). CG = no intervention	Moderate to severe change in heart rate. HF change. Change between HF and minutes of physical activity. Physical activity tolerance (IPAQ)	The moderate to severe heart failure decreased more in EG than in CG (difference of means: −2.7, 95% CI −4.2–−1.3). The mean percentage reduction was −43.6% (−56.0–−31.3) in EG versus no change in CG.
Hakestad, K.A. et al., 2015 [15]	Norway	Yes	N = 194 EG = 42 CG = 38	64.7	NR	Postmenopausal women with low BMD + fracture of healed wrist > 2 years	6 months	EG = ST + EQ CG = no intervention. Two group sessions and one at home (each 1 h) a week.	Quadricep strength (dynamometer). Body anthropometry (BMI). Absolute mass, % fat and BMD (DXA). Dynamic balance (FSST). Ability to walk (6 min walk test). Physical activity level (PASE). Quality of life (SF-36).	The means calculated for the EG improved or were stable during the follow-up, except for the reduction in right quadricep strength and the BMD in the lumbar spine and femoral trochanter. For CG, the mean values decreased during the follow-up, except for some of the variables for quadricep strength. There were no significant differences between the two groups during the 1-year follow-up for any of the outcome measures.
Hartley, C. et al., 2020 [22]	United Kingdom	Yes	N = 42 EG = 21 CG = 21	61.7	NR	Postmenopausal women ≥12 months amenorrhea	6 months	EG: EL CG: CL Both <15 min/day. One session/day.	BMD, BMC and femoral neck Z (DXA) Biochemical composition of the cartilage joint and associated disease with OA (magnetic resonance imaging—T2 relaxometry).	The BMD of the femoral neck, BMC and Z increased in EG (+0.81%, +0.69% and +3.18%, respectively) compared with CG (−0.57%, −0.71% and −0.75%: all interaction effects *p* < 0.05). There was no overall effect given that the mean T2 relaxation time (principal effect of time *p* = 0.011) did not differ between EL and CL.
Son, W.M. et al., 2020 [23]	South Korea	No	N = 20 EG = 10 CG = 10	67.7	NR	Postmenopausal women ≥12 months amenorrhea and stage 1 hypertension	12 weeks	EG = ERB CG = no intervention. Both three times/week for 60 min/session.	Values for estradiol, GH, IGF-1, DHEA-S (blood sample), BP (automatic sphygmomanometer), body mass, % body fat and lean body mass (anthropometry and bioelectrical impedance).	Significant group changes by time (*p* < 0.05) for estradiol, GH, IGF-1, DHEA-S and lean body mass, which increased significantly (*p* < 0.05), and systolic blood pressure, total body mass, body mass index and % body fat, which decreased significantly (*p* < 0.05) in EG, compared with no change in CG. There were no significant differences (*p* > 0.05) in diastolic blood pressure after 12 weeks.
Bittar, S.T. et al., 2015 [24]	Brazil	No	N = 60 EG = 30 CG = 30	67.3	G1 = 14 G2 = 12	Postmenopausal women >12 months	12 months	EG = SE (1 h, two times/week). CG = HE (two times/week).	BMD (DXA). Body composition, fat and lean mass (absorptiometry).	EG exhibited increased lean mass in the arms (*p* = 0.003) and legs (*p* = 0.011), total lean tissue (*p* = 0.015) and appendicular lean mass index (*p* = 0.001) compared with the baseline. CG showed no differences in lean mass.
Prado-Nunes, P.R. et al., 2016 [25]	Brazil	Yes	N = 32 CG= 13 EG1 = 13 EG2 = 12	61.3	6	Postmenopausal women >12 months. FSH >40 mUl/mL	16 weeks	CG = S (two times/week). EG1 = LV (three times/week). EG2 = HV (three times/week).	Leg extension strength (dynamometer). Body anthropometry (measuring tape, adipometer). Metabolic and inflammatory indicators (blood test).	EG2 experienced a reduction in total cholesterol and a smaller change in IL-6 compared with G1 (11.2% (P25–75, −7.6–28.4%) vs. 99.55% (P25–75 = 18.5–377.0%) for G3 and G1, respectively; *p* = 0.049) EG1 experienced a reduction in the percentage of HbA1c. There were positive correlations between WHR and IL-6 and between IL-6 and total cholesterol.
Martinho, N.M. et al., 2016 [26]	Brazil	Yes	N = 47 EG = 27 CG = 20	61.4	NR	Postmenopausal women >12 months	5 weeks	EG = RV. CG = M, S, ST and RE Both 30 min, two times/week.	SP strength (vaginal palpation and dynamometer)	There were no significant differences between the groups in most of the analyzed parameters. The muscle resistance was increased in EG (*p* = 0.003; effect size, 0.89; difference of means, 1.37; 95% CI 0.46–2.28).
Multanem, J. et al., 2017 [27]	Finland	No	N = 80 EG = 40 CG = 40	58.3	4	Postmenopausal women with knee pain in recent days	12 weeks	EG = leaps (three times/week, lasting 55 min). CG = no intervention.	Bone strength (femoral neck scanner). Knee cartilage (scanner). Physical activity (body movement monitors). Aerobic exercise load (number and intensity of leaps). Quality of life (SF-36).	Significant difference between the groups in the strength of the femoral neck flexion in favor of EG at 12 months (*p* < 0.01). The change in resistance to the femoral neck flexion remained significant after adjusting the baseline value, age, height and body mass (*p* = 0.020). In all participants, the change in resistance to flexion was associated with the total physical activity load (*p* = 0.012). EG had no effect on knee cartilage composition.
Ganderton, C. et al., 2018 [28]	Australia	Yes	N = 94 EG = 46 CG = 48	61. 8	NR	Postmenopausal women >52 weeks of amenorrhea with trochanteric pain	52 weeks	EG = strengthening exercise program. GLoBE + HE CG = e.g., simulated + HE Both 12 weeks	Gluteal tendon pain (VISA-G questionnaire). Perceived improvement in hip dysfunction (OHS and global rating of change). Quality of life (AQoL). Hip dysfunction and osteoarthritis (HOOS and lateral hip questionnaire).	EG achieved a significantly higher score in VISA-G, HOOS, OHS and lateral hip pain compared with CG. However, the intent-to-treat analysis showed no differences between the groups. There were no differences between the groups in the global rating of change at 12 (*p* = 0.340) and 52 weeks (*p* = 0.746). There was a significant improvement in the VISA-G score for both groups at 12 and 52 weeks (*p* < 0.001).
Watson, S.L. et al., 2018 [29]	Australia	No	N = 101 EG = 49 CG = 52	65	NR	Postmenopausal women with low bone mass	8 months	EG = W+ R CG = HE Both twice a week for 30 min.	Physical activity level (IPAQ). Daily calcium intake (AusCal). FN and LS BMD (DXA). Dominant or nondominant heels (QUS). Leg extension strength (dynamometer). Leg neuromuscular performance (maximum vertical leap test in a force plate).	Largest effects in EG for LS-BMD (2.9% and 2.8% vs. −1.2% and 2.8%, *p* < 0.001), FN-BMD (0.3% and 2.6% vs. −1.9% and 2.6%, *p* = 0.004), FN cortical thickness (13.6 16.6% vs. 6.3% and 16.6%, *p* = 0.014), height (0.2 and 0.5 cm versus −0.2, 0.2 and 0.5 cm *p* = 0.004) and all the performance measurements (*p* < 0.001).
Nunes, R.P.P. et al., 2019 [30]	Brazil	Yes	N = 38 CG = 13 EG1 = 13 EG2 = 12	60.9	NR	Postmenopausal women ≥12 month amenorrhea + FSH >40 mIU·mL^−1^ + E_2_ ≤54.7 pg·mL^−1^	16 weeks	CG = control. No RT, only S two times/week. EG1 = W+ LV + E; 45 min x three times/week. EG2 = W+ HV + E; 90 min x three times/week.	Strength performance (maximum strength test). Dietary intake E, CHO, LIP, PTN (food record). DHEA- S, TT, CO, TT:CO, IGF- 1 (blood sample).	EG1 and EG2 increased the performance of 1RM in all exercises compared with CG. There were no differences in strength performance between EG1 and EG2, except with the bar curl, which only increased in EG1. EG1 and EG2 increased the lean mass compared with CG (CG = 0.8 (95% CI 20.1–1.8) kg; EG1 = 2.2 (95% CI 1.7–2.7) kg; EG2 = 2.4 (95% CI 1.2–3.6) kg; p ANOVA = 0.037). There were no differences for the food intake and hormonal responses.
Watson, S.L. et al., 2019 [31]	Australia	NR	N = 51 EG = 25 CG = 26	64	NR	Postmenopausal women with low bone mass	8 months	EG = W+ R. CG = HE. Both twice a week for 30 min.	Magnitude of kyphosis (DXA, inclinometer and flexicurve)	EG obtained a high reduction in thoracic kyphosis in standing compared with G2 (−6.7 ± 8.2° vs. −1.6 ± 8.1°, *p* = 0.031). The two groups achieved an improvement within the group in kyphosis in the relaxed position, measured by the inclinometer and flexicurve (*p* < 0.05). There were no changes in the classification of vertebral fractures in EG after the intervention.

Abbreviations: AQoL, Assessment of Quality of Life; AusCal, Australian Calcium-focused Questionnaire; BMC, bone mineral content; BMD, bone mineral density; BMI, body mass index; BPAQ, Bone-Specific Physical Activity Questionnaire; BP, blood pressure; CG, control group; CHO, carbohydrate intake; CL, contralateral leg; CO, cortisol; DHEA-S, dehydroepiandrosterone sulfate; DXA, dual-energy X-ray absorptiometry; E, energy intake; E2, baseline estradiol; EG, experimental group; EG1, experimental group 1; EG2, experimental group 2; EL, exercise leg; ERB, exercises with resistance band; FN, femoral neck; FSH, follicle-stimulating hormone; EQ, equilibrium; FSST, Four Square Step Test; GH, growth hormone; HbA1c, hypercholesterolemia and borderline values of glycated hemoglobin; HE, home exercises; HF, hot flashes; HOOS, scoring of osteoarthritis result; HV, high volume; IGF-1, insulin-like growth factor-1; IL-6, interleukin-6; IPAQ, International Physical Activity Questionnaire; LIP, lipid intake; LS, lumbar spine; LV, low volume; M, mobility; N, number of participants; NR, Not Reported; OHS, Oxford Hip Score; PASS, Physical Activity Scale for the Elderly; PTN, protein intake; QUS, quantitative ultrasound; R, resistance; RE, relaxation; RT, resistance training; S, stretching; SE, supervised exercises; SF-36, health-related quality-of-life questionnaire; ST, strength; TT, testosterone; TT:CO, testosterone–cortisol ratio; VISA-G, gluteal tendon questionnaire; VR, virtual reality; W, warmup; WHR, waist-to-hip ratio; Z, section modulus.

**Table 2:** Characteristics of the Strength Exercise Interventions Included in this Review.

Study	Type	Material	Time/Session	Series	Repetitions	Repetition Time	Supervised
Berin, E. et al., 2019 [7]	C+ chest press, leg press, rowing, F leg, pulldown, E leg, abdominals and E of back + dynamic and static stretching	Physical fitness machines	225 min/week as the maximum (7–10 min of C+ stretching)	2	8–12	NR	Yes
Hakestad, K.A. et al., 2015 [15]	F, E and W exercises.	Weighted vest	60 min	NR	NR	NR	NR
Hartley, C. et al., 2020 [22]	W+ Leap with a single unilateral leg.	NR	8–9 min (C- 5 min + 3 or 4 min leap)	5 (15 s rest between series)	10	NR	Yes
Son, W.M. et al., 2020 [23]	W+ R (rowing, biceps curl, F shoulder, flexing, FE hip, heel raising, squats) + Cool down	Elastic band	60 min (40 min R)	1–4 weeks: 2–3 series (40–50% of 1RM). 5–8 weeks: 2–3 series (50–60% of 1RM). 9–12 weeks: 3–4 series (60–70% of 1RM).	1–4 weeks: 11–12 reps 5–8 weeks: 13–14 reps 9–12 weeks: 15–16 reps	NR	Yes
Bittar, S.T. et al., 2015 [24]	W+ impact exercises with fast walking with active movement of the arms and/or step exercises on a treadmill + R for arms, legs and abdomen + e.g., recreational exercises with a ball and a massage.	Elastic bands + improvised weights	60 min (10 min W, 20–25 min impact exercises with walking, 20–25 min R and 5 min, e.g., recreational exercises with a ball and a massage).	R—3	W (2 reps) R (10 reps)	W (20 s)	Yes
Prado- Nunes, P.R. et al., 2016 [25]	W+ Squats, F legs, E arms, bench press, rowing, pull down, triceps pulley and flexing with bar.	Physical fitness machines	NR	In W, 1 series exercise; in G1, 3 series (with 1.5 min between series) or in G3, 6 series.	W (15 reps) Exercise (8–12 reps)	W (40% of 1RM) Exercise (70% of 1RM)	Yes
Martinho, N.M. et al., 2016 [26]	Pelvic movements, maintaining control and trunk stabilization, with abdominal activation.	Virtual reality	30 min	4 (games)	1	5 min	Yes
Multanem, J. et al., 2017 [27]	Leap exercises with change in direction with music.	NR	55 min	NR	NR	NR	Yes
Ganderton, C. et al., 2018 [28]	Isometric load gluteus medius and minimus + strengthening of quadriceps and calves.	Chair as a safety measure	NR	2–4	5–15	40 s	Yes
Watson, S.L. et al., 2018 [29]	W (dead weight) + R (dead weight, air pressure and squats)	Physical fitness machines	30 min	W—2 R—5	W—5 (50–70% of 1RM) W—5 (> 80–85% of 1RM)	NR	Yes
Nunes, R.P.P. et al., 2019 [30]	W+ squats, leg curl, leg extension, bench press, rowing, pull down, bar curl, triceps pulley + S	Physical fitness machines	EG1 = 45 min EG2 = 90 min.	W = 1 series (40% of 1RM). EG1 = 3 series (70% of 1RM). EG2 = 6 series (70% of 1RM).	W = 15 reps. EG1 = 8–12 reps. EG2 = 8–12 reps.	NR	Yes
Watson, S.L. et al., 2019 [31]	R progressive (low load, dead weight, bench press and back squats) + CI (dominated with leaps)	Physical fitness machines	30 min	5	5 (80–85% of 1RM)	NR	Yes

Abbreviations: E, extension; EG1, experimental group 1; EG2, experimental group 2; F, flexion; FE, flexion-extension; NR, not reported; R, resistance; RM, repetition maximum; S, stretching; W, warm up.

**Table 3:** Risk-of-bias summary: review authors’ judgements about each risk-of-bias item for each included study.

Study	Criteria							
	1	2	3	4	5	6	7	8
Berin, E. et al., 2019 [7]	Low	Low	High	Low	Low	Low	Low	Low
Hakestad, K.A. et al., 2015 [15]	Low	Low	Low	Low	Low	Low	Low	Low
Hartley, C. et al., 2020 [22]	High	Low	Low	Low	Low	Low	Low	Low
Son, W.M. et al., 2020 [23]	Low	Low	Low	High	Low	Low	Low	Low
Bittar, S.T. et al., 2015 [24]	Low	High	High	High	Low	Low	Low	Unclear
Prado- Nunes, P.R. et al., 2016 [25]	High	High	High	High	Low	Low	Low	High
Martinho, N.M. et al., 2016 [26]	Low	Low	Low	High	Low	Low	Low	Low
Multanem, J. et al., 2017 [27]	Low	High	Low	High	Low	Low	Low	Low
Ganderton, C. et al., 2018 [28]	Low	High	Low	Low	Low	Low	Low	Low
Watson, S.L. et al., 2018 [29]	Low	Low	High	High	Low	Low	Low	Unclear
Nunes, R.P.P. et al., 2019 [30]	High	High	High	High	Low	Low	Low	High
Watson, S.L. et al., 2019 [31]	Low	Low	High	High	High	Low	Low	Unclear

Data extracted from the Cochrane Risk-of-Bias Tool. Criteria: 1, Random sequence generation; 2, Allocation concealment; 3, Blinding of participants and personnel; 4, Blinding of outcome assessment; 5, Incomplete outcome data; 6, Selective reporting; 7, Other bias; 8, Risk of bias.

## Data Availability

Not applicable.
